# Resolving Host-Associated Antimicrobial Resistance in Municipal Wastewater Using Hi-C Metagenomics

**DOI:** 10.3390/microorganisms14102153

**Published:** 2026-09-24

**Authors:** Abiola Senok, Subham Verma, Douha Shouqair, Rashed Alghafri, Danesh Moradigaravand, Rania Nassar, Dean Everett, Richard Goering, Mushtaq Khan

**Affiliations:** 1 College of Medicine, Mohammed Bin Rashid University of Medicine and Health Sciences, Dubai P.O. Box 505055, United Arab Emiratesrania.nassar@dubaihealth.ae (R.N.); 2 Center for Microbial Sciences, Mohammed Bin Rashid University of Medicine and Health Sciences, Dubai P.O. Box 505055, United Arab Emirates; 3 School of Dentistry, Cardiff University, Cardiff CF14 4XY, UK; 4 General Department of Forensic Science and Criminology, Dubai Police, Dubai P.O. Box 1493, United Arab Emirates; r.alghafri@dubaipolice.gov.ae; 5 Laboratory of Infectious Disease Epidemiology, Biomedical Sciences (BioMed) Division, King Abdullah University of Science and Technology (KAUST), Thuwal 23955-6900, Saudi Arabia; 6 Department of Public Health and Epidemiology, College of Medicine and Health Sciences, Khalifa University, Abu Dhabi P.O. Box 127788, United Arab Emirates; dean.everett@ku.ac.ae; 7 Biotechnology Center, Khalifa University, Abu Dhabi P.O. Box 127788, United Arab Emirates; 8 Infection Research Unit, Khalifa University, Abu Dhabi P.O. Box 127788, United Arab Emirates; 9 Department of Medical Microbiology and Immunology, Creighton University School of Medicine, Omaha, NE 68178, USA; 10 Department of Medical Microbiology and Immunology, College of Medicine and Health Sciences, United Arab Emirates University, Al Ain P.O. Box 15551, United Arab Emirates; 11 Zayed Center for Health Sciences, United Arab Emirates University, Al Ain P.O. Box 15551, United Arab Emirates

**Keywords:** antimicrobial resistance, Hi-C metagenomics, municipal wastewater, One Health surveillance

## Abstract

Conventional wastewater metagenomics enables population-level surveillance of antimicrobial resistance (AMR) but provides limited insight into the bacterial hosts, genomic context, and the mobility of resistance determinants. In this study, we applied high-throughput chromosome conformation capture (Hi-C) metagenomics, a proximity-ligation approach, to resolve host-associated AMR. Six influent wastewater samples collected from two municipal wastewater treatment plants over a four-month sampling period were investigated. Integrated shotgun metagenomic and Hi-C sequencing were used to reconstruct metagenome-assembled genomes (MAGs), plasmid bins, and viral bins, and to link AMR, stress-response, and virulence-associated genes to their bacterial hosts through Hi-C contacts. From 12,877 genome bins, 454 met quality thresholds (139 MAGs, 130 plasmid bins, 185 viral bins) spanning 70 bacterial and archaeal families. AMRFinderPlus identified 771 resistance and fitness-associated gene hits (517 AMR, 232 stress-response, 22 virulence-associated), dominated by macrolide–lincosamide–streptogramin B (*n* = 117), β-lactam (*n* = 114), and tetracycline (*n* = 96) determinants. Hi-C linked 490 gene hits to host families; Enterobacteriaceae had the highest number of host-assigned detections (*n* = 131), while Aeromonadaceae carried the broadest AMR repertoire (*n* = 102; 72% AMR). The host–plasmid network comprised 137 AMR-positive plasmid bins linked to 106 MAGs through 166 Hi-C-supported associations. Five plasmid contigs, including one harbouring *bla*_CMY-4_, were linked to MAGs from two distinct bacterial classes, suggestive of cross-lineage plasmid carriage. Viral AMR attribution was limited, with *erm(B)* as the sole resistance determinant identified on a viral contig. Hi-C metagenomics extends wastewater resistome profiling beyond gene inventories by resolving bacterial hosts, plasmid associations, and genomic context, strengthening the ecological interpretation of wastewater-based AMR surveillance.

## 1. Introduction

Antimicrobial resistance (AMR) is one of the most pressing global health challenges, responsible for substantial morbidity and mortality worldwide and projected to increase in the absence of effective intervention [1,2,3]. The interconnected movement of resistance determinants among human, animal, and environmental compartments has firmly established AMR as a One Health challenge. Within this framework, the environment is increasingly recognized as an important reservoir where resistance determinants persist, evolve, and are exchanged among diverse microbial communities [4].

Shotgun metagenomic sequencing is a key method for characterizing wastewater resistomes, enabling culture-independent detection of antimicrobial resistance genes (ARGs) across complex microbial communities [5]. However, shotgun metagenomic analyses generally provide inventories of ARGs and their relative abundances without establishing which organisms harbour specific genes, whether those genes reside on chromosomes or mobile genetic elements, or the extent to which they move across taxonomic boundaries [6]. This gene–host–mobility linkage gap limits interpretation, as the public-health significance of an ARG depends not only on its presence but also on its host range and potential for horizontal transfer into clinically relevant pathogens [7]. Resolving these relationships is therefore essential for translating environmental resistome data into meaningful assessments of AMR risk. High-throughput chromosome conformation capture (Hi-C) metagenomics, a proximity-ligation approach, overcomes this gene–host–mobility linkage gap by capturing intracellular DNA contacts and enabling the assignment of plasmids, bacteriophages, and ARGs to their microbial hosts within complex communities [8]. This capability is particularly relevant to AMR surveillance, as horizontal gene transfer mediated by mobile genetic elements is a major driver of resistance dissemination [9,10]. Although Hi-C has been applied to microbiome studies and selected environmental systems, its use for host-resolved investigation of wastewater resistomes remains limited [11,12,13], and data from the Arabian Gulf region are lacking. This study was carried out to address this gap in the literature by using integrated Hi-C with shotgun metagenomics to resolve the bacterial hosts and genomic context of ARGs within wastewater microbial communities and to investigate the ecological organization of the resistome. By moving beyond gene inventories, this approach advances environmental AMR surveillance and provides new insight into resistance ecology at the human–environment interface.

## 2. Materials and Methods

### 2.1. Wastewater Sampling and DNA Extraction

To provide coverage of the municipal wastewater system, influent samples were collected monthly, between October 2024 and January 2025, from the two wastewater treatment plants (WWTPs) serving Dubai, United Arab Emirates. Twenty-four-hour composite WWTP influent samples were collected using refrigerated autosamplers (Hach, Loveland, CO, USA). For each sampling event, 1 L of wastewater was collected and transported to the laboratory at 4 °C. A 14 mL aliquot of wastewater was centrifuged at 7500 rpm for 10 min to pellet microbial biomass. The pellet was resuspended in PG Shield (Phase Genomics, Seattle, WA, USA), and total community DNA was extracted using the DNeasy PowerSoil Pro Kit (QIAGEN, Hilden, Germany) according to the manufacturer’s instructions. DNA concentration was determined using a Qubit Flex fluorometer and the Qubit dsDNA High Sensitivity Assay Kit (Thermo Fisher Scientific, Waltham, MA, USA).

### 2.2. Shotgun Metagenomic and Hi-C Sequencing

Shotgun metagenomic libraries were generated using the Watchmaker DNA Library Prep Kit (Watchmaker Genomics, Boulder, CO, USA) according to the manufacturer’s instructions. Following platform conversion using the Adept workflow (Element Biosciences, San Diego, CA, USA), libraries were sequenced on an Element AVITI platform using paired-end 150 bp chemistry [14].

Hi-C libraries were generated using the ProxiMeta Hi-C Kit v4.0 (Phase Genomics, Seattle, WA, USA) according to the manufacturer’s protocol [15]. Intact microbial cells were formaldehyde-crosslinked, digested with Sau3AI and MluCI restriction enzymes, and proximity-ligated to capture intracellular DNA associations. Libraries were sequenced on an Illumina NovaSeq platform using paired-end 150 bp reads [16].

### 2.3. Assembly, Hi-C Deconvolution and Taxonomic Classification

Shotgun metagenomic reads were quality-filtered using fastp v0.20.1 with default parameters (minimum Phred quality score Q15, automatic adapter trimming, minimum read length 15 bp) and assembled de novo with MEGAHIT v1.2.9 using default k-mer parameters (k-min = 21, k-max = 141, k-step = 12). Hi-C reads were aligned to assembled contigs using BWA-MEM v0.1.17 with the -5SP option, followed by duplicate removal with SAMBLASTER v0.1.24. Alignments were filtered with SAMtools v1.9 using the -F 2304 flag to remove non-primary and secondary alignments. Metagenome deconvolution was performed with the ProxiMeta (Phase Genomics) platform [17,18], which clusters assembled contigs into metagenome-assembled genomes (MAGs) based on Hi-C proximity-ligation linkage. MAG quality was assessed using CheckM v1.1.3. Taxonomic assignments were performed using GTDB-Tk v2.4.0 with GTDB release v220. For downstream analyses, microbial MAGs with ≥70% completeness and ≤10% contamination were retained as high quality.

### 2.4. Identification of Plasmids, Viruses and Host Attribution

Viral contig and viral MAG (vMAG) quality was assessed using CheckV v1.0.1 [19]. Putative plasmid contigs were identified by blastn comparison of all assembled contigs against a custom version of the PLSDB plasmid database (v2020_06_29; [20]), applying the following thresholds: ≥90% sequence identity, minimum alignment length of 100 bp, ≥50% of the query contig length aligned to the reference plasmid, and ≥50% of the reference plasmid covered by the query contig. Host assignment for both viral and plasmid bins was performed using the ProxiPhage host attribution tool [15,21], which leverages long-range Hi-C linkages between extrachromosomal elements and prokaryotic host genomes. Viral–host and plasmid–host linkages were filtered on Hi-C linkage strength, connectivity ratios, and intra-MAG connectivity. Associations were retained if they met all of the following: ≥2 Hi-C read links, connectivity ratio ≥0.1, and a copy number threshold derived from a ROC curve optimized to maximize host-assigned elements while minimizing false-positive linkages. Connections with average copy count <80% of the per-element maximum were additionally removed to reduce spurious assignments. Only host associations involving microbial MAGs meeting quality thresholds (≥70% completeness and ≤10% contamination) were retained. For comparative analyses, plasmid and viral bins were required to meet minimum quality thresholds of ≥50% completeness and ≤20% contamination. Microbial MAG thresholds were selected above the MIMAG medium-quality standard [22]. Plasmid and viral bin thresholds reflect the structural diversity and inherent fragmentation of extrachromosomal elements and are consistent with MIUVIG medium-quality criteria [23].

### 2.5. Antimicrobial Resistance and Network Analyses

AMR, stress-response, and virulence-associated genes were identified using AMRFinderPlus v3.10.5 with the plus option enabled and default parameters. Gene annotations were integrated with Hi-C host attribution data to assign ARGs, virulence determinants, and stress-response genes to microbial MAGs, plasmid bins, and viral bins and to determine their genomic context. For resistome analyses, macrolide-, lincosamide-, and streptogramin-associated resistance determinants were grouped into a single MLSB category. Rare composite resistance classes were consolidated into an “Other” category for visualization. Host–plasmid association networks and gene-centred AMR interaction networks were constructed by integrating Hi-C linkage data with plasmid and AMRFinderPlus annotations, enabling characterization of the relationships among microbial hosts, mobile genetic elements, and resistance determinants. Community composition analyses, heatmaps, and network visualizations were generated in Python v3.13.9 using Pandas v2.3.3, NumPy v2.4.0, Seaborn v0.13.2, and Matplotlib v3.10.8.

## 3. Results

### 3.1. Hi-C Metagenomic Deconvolution Recovers Microbial, Plasmid and Viral Genome Bins from Wastewater Inflows

A total of eight wastewater influent samples (four each from WWTP1 and WWTP2) were obtained during the sample collection period. Two samples from WWTP1 failed Hi-C sequencing and were excluded from further downstream analysis. For one sample, Hi-C reads mapped poorly to the assembly generated from the corresponding shotgun metagenomic reads, precluding reliable downstream deconvolution. For the second sample, two independent attempts to generate Hi-C libraries resulted in insufficient sequencing yield. Across the remaining six samples, 12,877 genome bins were recovered (microbial: *n* = 1087; plasmids: *n* = 8841; viral: *n* = 2943) and there were six contigs which remained unbinned (Table 1). The total binned assembly length was 4.32 Gb, supported by 2.59 × 10^8^ shotgun reads mapped to binned contigs. Bin recovery scaled with sequencing yield and was substantially higher in the two samples from WWTP-1 (2677 and 4380 bins) than in any individual sample from WWTP-2 (range 721–1972 bins). Applying the class-specific quality thresholds (see methods), a total of 454 bins comprising 139 microbial MAGs, 130 plasmid bins, and 185 viral bins were retained across all samples (Table 1; Figure 1). Of the 185 retained viral bins, 42 were assigned to the CheckV Complete tier (closed-genome confidence) and a further 17 to the high-quality tier (≥90% completeness).

The unfiltered microbial bin pool spanned the full spectrum of assembly quality, with median CheckM completeness, contamination, N50 and assembly length being 10.3%, 0.0%, 2072 bp, and 555 kb, respectively. Every sample yielded numerous MAGs meeting the quality thresholds, with WWTP-1-Jan (*n* = 39) and WWTP-1-Dec (*n* = 31) contributing the greatest numbers. Across the unfiltered dataset, plasmid bins outnumbered microbial bins by 8.1-fold and were considerably shorter (median length, 1418 bp; median GC content, 54.0%) than viral bins (median length, 7666 bp). Of the 2943 unfiltered viral bins (Table 1), 69 (2.3%) were classified as integrated proviruses.

### 3.2. Taxonomic Composition of Microbial MAGS, Plasmid and Viral Bins

To compare taxonomic composition among microbial MAGs, plasmid and viral bins, the quality-filtered bins were aggregated at the GTDB family level and visualized for each sample (Figure 2). The 139 quality-filtered microbial MAGs were distributed across 70 bacterial and archaeal families. Burkholderiaceae and Pasteurellaceae were the most abundant families (*n* = 9 each), followed by Aeromonadaceae and Bifidobacteriaceae (*n* = 7 each), Veillonellaceae (*n* = 6) and Peptostreptococcaceae (*n* = 5). The 15 most abundant families are shown individually in Figure 2A; the remaining 55 families collectively accounted for 46.8% of recovered MAGs and were grouped as ‘Other families’. Family composition was diverse and varied among samples, with no single family dominating across all sites. Veillonellaceae was detected in five of the six samples. Of the 130 quality-filtered plasmid bins, 91 (70.0%) were assigned to bacterial host families (Figure 2B). Enterobacteriaceae dominated the plasmid-associated host families, accounting for 55 bins (42.3%) and occurring in all samples except WWTP-1-Jan. Bifidobacteriaceae was the second most abundant host family (*n* = 11), whereas Aeromonadaceae, Streptococcaceae, Bacteroidaceae and Ruminococcaceae each contributed smaller proportions of the plasmidome (Figure 2B). Two plasmid bins were linked to multiple bacterial host families, including one associated with both Bifidobacteriaceae and Enterobacteriaceae (Figure 2B). Host assignment was substantially lower for viral bins. Out of the 185 quality-filtered viral bins, 11 (5.9%) were assigned to bacterial host families, whereas 174 (94.1%) remained unassigned (Figure 2C). The host-assigned viral bins were distributed across ten bacterial families, of which only Moraxellaceae was associated with more than one viral bin. Despite marked differences in host-assignment rates, consistent taxonomic patterns were evident across microbial MAGs, host-assigned plasmid bins, and viral bins. Enterobacteriaceae and Aeromonadaceae were identified in both microbial MAGs and plasmid-associated host families, highlighting these taxa as major reservoirs of mobile genetic elements in wastewater.

### 3.3. Resistome Composition and Host Distribution of Antimicrobial Resistance, Stress-Response and Virulence Determinants

Using AMRFinderPlus, 771 resistance- and fitness-associated gene hits were identified, comprising 517 AMR genes, 232 stress-response genes, and 22 virulence-associated genes. Following harmonization of closely related resistance categories, these resolved into 31 gene classes comprising 16 AMR, 14 stress-response, and one virulence-associated (Figure 3A). The resistome was dominated by MLSB resistance determinants (*n* = 117) and β-lactam resistance genes (*n* = 114), followed by tetracycline (*n* = 96), aminoglycoside (*n* = 65), phenicol (*n* = 32) and quinolone (*n* = 26) resistance determinants. Sulfonamide (*n* = 17), trimethoprim (*n* = 15), and efflux-associated genes (*n* = 13) occurred less frequently, whereas glycopeptide, rifamycin, colistin, streptothricin, fosfomycin, and nitroimidazole resistance genes were rare (≤6 hits per class).

Stress-response genes were dominated by heavy-metal resistance determinants. Mercury resistance genes were most abundant (*n* = 73), followed by copper (*n* = 36), arsenic (*n* = 28) and copper/silver resistance genes (*n* = 26). Together, these four categories accounted for approximately 70% of stress-response gene hits. Heat-response genes (*n* = 22) and genes conferring resistance to quaternary ammonium compounds (*n* = 17) comprised most of the remaining stress-response genes. Virulence-associated genes were infrequent (*n* = 22) and were detected exclusively in the WWTP-1-Dec sample.

The genomic distribution of resistance-associated genes differed across contig types. Stress-response genes showed the highest plasmid association, with 58.6% (*n*/*N* = 136/232) located on plasmid contigs, compared with 39.5% (*n*/*N* = 204/517) of AMR genes and 40.9% (*n*/*N* = 9/22) of virulence-associated genes. Among stress-response genes, plasmid localization was most frequent for arsenic (71%; *n*/*N* = 20/28), copper (67%; *n*/*N* = 24/36), mercury (64%; *n*/*N* = 47/73), and copper/silver resistance genes (58%; *n*/*N* = 15/26). Among AMR genes, sulfonamide (65%; *n*/*N* = 11/17), colistin (60%; *n*/*N* = 3/5), quinolone (58%; *n*/*N* = 15/26) and tetracycline (56%; *n*/*N* = 54/96) resistance determinants were more frequently detected on plasmids than chromosomal contigs. In contrast, glycopeptide (100%; *n*/*N* = 6/6), efflux-associated (77%; *n*/*N* = 10/13), aminoglycoside (51%; *n*/*N* = 33/65), and β-lactam (46%; *n*/*N* = 53/114) resistance genes were more frequently detected on chromosomal contigs. A single *erm(B)* gene was identified on a viral contig assigned to a *Streptococcus*-associated host bin.

Hi-C host assignment linked 490 gene hits (AMR, *n* = 323; stress-response, *n* = 155; virulence-associated, *n* = 12) to bacterial host families (Figure 3B). Enterobacteriaceae contained the largest number of host-assigned gene hits (*n* = 131), comprising 86 stress-response genes (66%), 34 AMR genes (26%) and 11 virulence-associated genes (8%). Aeromonadaceae contained 102 host-assigned gene hits, including 73 AMR genes (72%) spanning β-lactam, aminoglycoside, tetracycline, quinolone, trimethoprim and sulfonamide resistance classes, together with 29 mercury-resistance genes (28%). Burkholderiaceae contained 42 host-assigned gene hits, comprising 26 stress-response genes (62%), predominantly mercury resistance genes, and 16 AMR genes (38%). Seven additional families exceeded 10 host-assigned gene hits: Rhodocyclaceae (*n* = 18), Bacteroidaceae (*n* = 15), Peptostreptococcaceae (*n* = 15), Moraxellaceae (*n* = 14), Neisseriaceae (*n* = 14), Pseudomonadaceae (*n* = 14) and Streptococcaceae (*n* = 11). Peptostreptococcaceae and Streptococcaceae contained only AMR genes, whereas Pseudomonadaceae contained a single virulence-associated gene.

### 3.4. Hi-C Host–Plasmid Association Network of AMR-Positive Plasmids

Reconstruction of Hi-C host–plasmid associations yielded 137 AMR-positive plasmid bins linked to 106 microbial MAGs through 166 Hi-C-supported associations. The resulting network comprised 79 connected components. Most plasmid bins showed low host connectivity (mean degree, 1.21; maximum, 3), whereas microbial MAGs exhibited greater connectivity (mean degree, 1.57; maximum, 10).

Aeromonadaceae accounted for the largest number of host–plasmid associations (30 associations involving 28 AMR-positive plasmid bins), followed by Enterobacteriaceae (19 associations involving 17 plasmid bins) (Table 2). Bifidobacteriaceae, Rhodocyclaceae, Neisseriaceae, Peptostreptococcaceae, and Pseudomonadaceae each accounted for smaller numbers of host–plasmid associations (Table 2).

The highest-degree microbial MAGs were a *Klebsiella* MAG from WWTP-1-Dec and a *Tolumonas* MAG from WWTP-2-Jan, each linked to ten AMR-positive plasmid bins. Other microbial MAGs with higher degrees included *Tolumonas* MAGs from WWTP-2-Nov and WWTP-1-Dec (degrees 8 and 4), a *Neisseria* MAG from WWTP-2-Dec (degree 6), and *Pseudomonas*, *Escherichia* and *Propionivibrio* MAGs with lower degrees of connectivity. No plasmid bin was associated with more than three microbial MAGs. Five plasmid contigs were linked to MAGs belonging to two bacterial classes within the same sample (Supplementary Table S1). These included contig k99_1341003 carrying *bla*_CMY-4_, linked to both Clostridia and Synergistia MAGs in WWTP-1-Dec, and two contigs from WWTP-2-Nov: contig k99_420566 carrying *aph(3″)-Ib* and *aph(6)-Id*, and contig k99_152708 carrying *tet(C)* each linked to both Gammaproteobacteria and Alphaproteobacteria MAGs. All five contigs were classified as plasmid-derived. In addition, *erm(B)* was identified on a viral contig assigned to a *Streptococcus*-associated MAG in WWTP-2-Nov.

### 3.5. Gene-Centred Host–AMR Association Network

A gene-centred network was constructed from the 30 most abundant AMR genes detected across all samples after excluding unclassified and host-unresolved assignments (Figure 4). The network comprised 144 host-linked gene detections, including 86 plasmid-associated (59.7%), 57 chromosomal (39.6%) and one viral-associated detection. Tetracycline resistance genes and MLSB resistance determinants each accounted for 37 detections, of which 26 (70%) and 22 (59%), respectively, were plasmid-associated (Figure 4). Aminoglycoside resistance determinants accounted for 16 detections, of which 11 (69%) were plasmid-associated. The remaining resistance determinants showed more variable genomic distributions. Sulfonamide (89%; 8/9), quinolone (80%; 4/5), trimethoprim (100%; 4/4), macrolide *mph(A)* (100%; 5/5), and β-lactamase *bla*_CMY-4_ (75%; 3/4) determinants were largely plasmid-associated, although *bla*_OXA_ was predominantly chromosomal (86%; 6/7). The glycopeptide-associated regulator *vanR-O* (100%; 5/5) and the efflux pump gene *emrD* (100%; 7/7) were detected exclusively on chromosomal contigs.

Host-class analysis assigned 66 of 144 host-linked gene detections (45.8%) to Gammaproteobacteria, including 44 (67%) plasmid-associated detections. Host-linked gene detections assigned to Gammaproteobacteria included β-lactam, tetracycline, quinolone, aminoglycoside, sulfonamide, macrolide and efflux-associated resistance determinants. Additional host-linked detections were assigned to Clostridia (*n* = 19; 13.2%), Actinomycetes (*n* = 14; 9.7%), Negativicutes (*n* = 14; 9.7%), Alphaproteobacteria (*n* = 8; 5.6%) and Bacteroidia (*n* = 7; 4.9%). Bacilli, Synergistia, Campylobacteria, Coriobacteriia and Thermoleophilia collectively accounted for the remaining 16 detections (11.1%).

## 4. Discussion

Wastewater-based shotgun metagenomics has transformed AMR surveillance by enabling culture-independent characterization of resistance genes at a population scale [24,25,26]. However, current surveillance frameworks using shotgun metagenomic sequencing remain largely gene-centric, describing the composition and abundance of ARGs while providing limited insight into their bacterial hosts, genomic context or association with mobile genetic elements [24,25,27]. Yet the ecological significance of an ARG is determined not only by its abundance but also by the bacterial population in which it resides, its genomic location, and its potential for persistence and dissemination within microbial communities. By applying Hi-C metagenomics, the present study addresses this gap by resolving the bacterial hosts and the genomic context of ARGs, advancing from descriptive resistome profiling towards a more ecologically informed understanding of AMR.

Previous studies have demonstrated the ability of Hi-C metagenomics to reconstruct in situ associations between bacterial hosts, ARGs, and mobile genetic elements in complex microbial communities, including wastewater [11,12,13], thereby establishing the foundation for host-resolved resistome analyses [16,23,28,29]. Building on this foundation, we applied Hi-C metagenomics across longitudinally collected wastewater samples to resolve host genomes, plasmids, viral elements, ARGs and stress-response determinants within a single analytical framework. This extended host-resolved analysis beyond individual communities towards an ecological understanding of the wastewater resistome. This integrated perspective has important implications for environmental AMR surveillance because wastewater samples with comparable resistome profiles may differ substantially in their ecological organization. These ecological differences, which are not captured by abundance-based profiling alone, may influence the persistence and dissemination potential of resistance within microbial communities [30]. Host attribution revealed marked functional partitioning among dominant bacterial families within the wastewater microbiome. Enterobacteriaceae accounted for the largest number of host-associated gene detections but were enriched predominantly for stress-response determinants, particularly heavy-metal resistance genes. Aeromonadaceae harboured the broadest diversity of AMR determinants across multiple drug classes, whereas Burkholderiaceae similarly exhibited enrichment for stress-response determinants. These contrasting profiles indicate that dominant bacterial taxa play distinct ecological roles, with some primarily contributing to environmental adaptation and others disproportionately associated with AMR. The prominence of Aeromonadaceae is consistent with the seminal Hi-C wastewater study by Stalder et al. [16], which identified members of this family as major hosts of ARGs linked to broad-host-range plasmids. The concordance between the two studies strengthens the evidence that Aeromonadaceae represent an important environmental component of the wastewater resistome. More broadly, these findings underscore the value of wastewater as a One Health surveillance matrix by capturing microbial populations from interconnected human, environmental, and engineered ecosystems, including taxa and associated resistance determinants that are likely to be underrepresented in isolate-based clinical surveillance.

Building on these host-level observations, reconstruction of the host–plasmid association network provided further insight into the ecological organization of the wastewater mobilome. Most AMR-positive plasmids exhibited limited host connectivity, whereas a comparatively small number of microbial genomes were linked to multiple plasmids, generating an asymmetrical network architecture. This pattern suggests that mobile resistance within these wastewater communities was organized primarily around bacterial hosts carrying multiple AMR-associated plasmids, rather than widespread dissemination of individual plasmids across unrelated taxa. Inspection of individual Hi-C-resolved plasmid contigs identified five instances in which a single plasmid contig carried contacts to MAGs from two distinct bacterial classes within the same sample (Supplementary Table S1), consistent with shared plasmid carriage across lineages. The most clinically significant of these involved contig k99_1341003 carrying *bla*_CMY-4_, an AmpC-type β-lactamase conferring resistance to third-generation cephalosporins, which was associated with both Clostridia and Synergistia MAGs. AmpC β-lactamases such as *bla*_CMY-4_ are of particular concern because they confer resistance to extended-spectrum cephalosporins and are refractory to inhibition by clinically used β-lactamase inhibitors, making their carriage on putatively mobile elements in environmental reservoirs epidemiologically significant [31]. The remaining four shared-contig events involving aminoglycoside resistance genes (*aph(3″)-Ib*; *aph(6)-Id*) and the tetracycline resistance gene *tet(C)* across Gammaproteobacteria, Alphaproteobacteria and Campylobacteria (Supplementary Table S1). Whilst these observations are consistent with shared plasmid carriage, Hi-C does not directly measure horizontal gene transfer. Rather, it resolves the genomic context of resistance by distinguishing chromosomal from mobile genetic element-associated determinants, and it complements conventional resistome profiling by resolving host–gene–mobility relationships. Accordingly, the reconstructed host–plasmid network identifies bacterial populations in which multiple resistance plasmids coexist, providing ecological insight beyond ARG abundance or plasmid inventories, but does not permit inference of the timing, frequency, or directionality of plasmid transfer. Although Hi-C-based host assignments represent proximity-ligation-supported associations, their reliability depends on linkage strength and the filtering criteria applied. Stringent thresholds were used in this study to reduce spurious associations; however, selected host–plasmid–ARG linkages were not independently validated using targeted PCR, long-read sequencing, or other orthogonal approaches. Such validation is recommended in future studies.

In contrast to plasmids, viral host attribution was limited, with only a single AMR determinant, *erm(B)*, identified on a viral contig, providing limited evidence of phage-associated AMR within the reconstructed wastewater mobilome. This finding should be interpreted cautiously given the recognized challenges of environmental viral metagenomics [15,26,32]. Partial viral host assignment has been previously reported [33], and this may reflect insufficient proximity-ligation signal from extracellular or low-abundance viral populations, fragmented or low-coverage viral assemblies, and incomplete representation of corresponding bacterial hosts within the recovered MAG set. These factors may have contributed to the low proportion of host-assigned viral bins observed in this study.

Resolving the genomic context of resistance determinants extended host attribution by distinguishing chromosomal from plasmid-associated resistance. Tetracycline and MLSB resistance determinants formed the principal plasmid-associated components of the reconstructed resistome, while sulfonamide, quinolone, trimethoprim and selected β-lactam resistance determinants were likewise frequently plasmid-associated. In contrast, *bla*_OXA_, *vanR-O* and *emrD* occurred predominantly or exclusively within chromosomal contexts. These contrasting distributions highlight that resistance burden and mobility potential are distinct properties of the wastewater resistome, with genes detected at similar frequencies differing substantially in their potential for dissemination according to genomic location. Collectively, these findings show that integrating host attribution with genomic context provides a more informative framework for interpreting environmental AMR. Stress-response determinants extended this ecological framework. Mercury, copper, arsenic, and copper/silver resistance genes dominated the stress-response repertoire and exhibited stronger plasmid association than ARGs overall. Heavy metals are persistent environmental contaminants that have long been implicated in maintaining AMR through co-selection, particularly where metal- and antibiotic-resistance determinants occur on shared mobile genetic elements [34,35,36]. Whilst the concurrent plasmid association of both stress-response and resistance determinants is compatible with this ecological model, physical co-localization of individual metal- and antibiotic-resistance genes on the same mobile genetic elements was not established, nor was co-selection demonstrated experimentally. Nevertheless, our findings support an ecological framework in which environmental selective pressures contribute to maintaining adaptive mobile genetic elements within wastewater communities.

A limitation of this study is the small sample size, compounded by the technical exclusion of two samples, which further reduced the analyzable dataset and representation of WWTP-1. MAG and bin recovery is also inherently threshold-dependent, and this is a limitation as more stringent quality criteria would reduce genome retention, particularly in lower-depth samples, and may consequently constrain the breadth of host-attribution analyses. Future studies incorporating deeper sequencing and larger, temporally representative sample sets would enable more robust sensitivity assessment and strengthen the generalizability of these findings.

## 5. Conclusions

In conclusion, this study highlights the potential of Hi-C metagenomics to extend wastewater AMR surveillance beyond resistance gene inventories by linking resistance determinants to bacterial hosts and genomic context. The findings provide ecological context for the organization of the wastewater resistome and its association with mobile genetic elements, while supporting further evaluation of host-resolved metagenomics as a complementary approach for wastewater AMR surveillance.

## Figures and Tables

**Figure 1:**
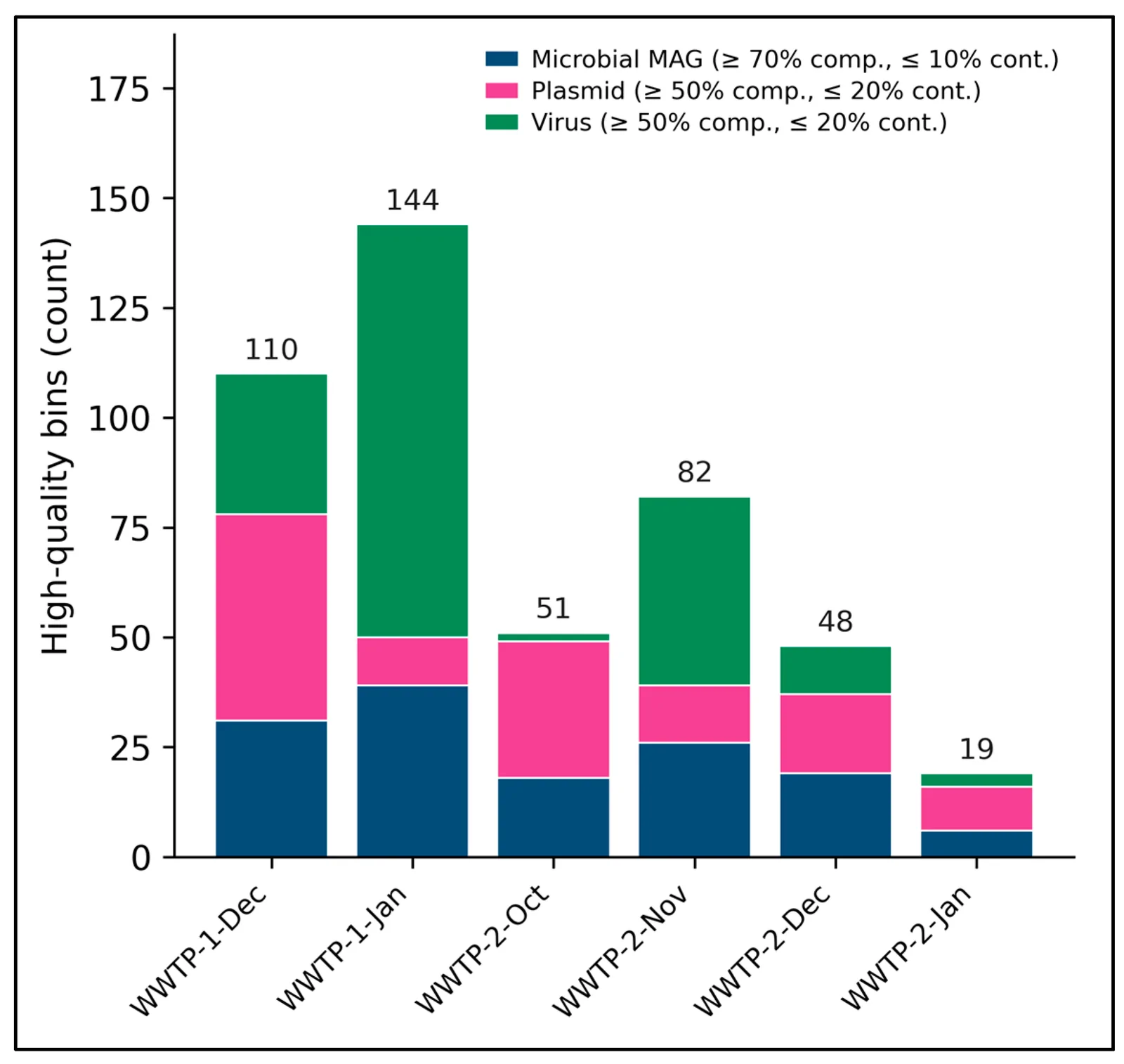
Hi-C metagenomic deconvolution of wastewater inlet samples. Stacked bar plot of high-quality bins recovered per sample, partitioned by genome category: microbial MAGs (≥70% CheckM completeness, ≤10% contamination), plasmid bins (≥50% ProxiMeta plasmid completeness, ≤20% contamination) and viral bins (≥50% CheckV completeness, ≤20% contamination). Total high-quality bin counts are annotated above each bar.

**Figure 2:**
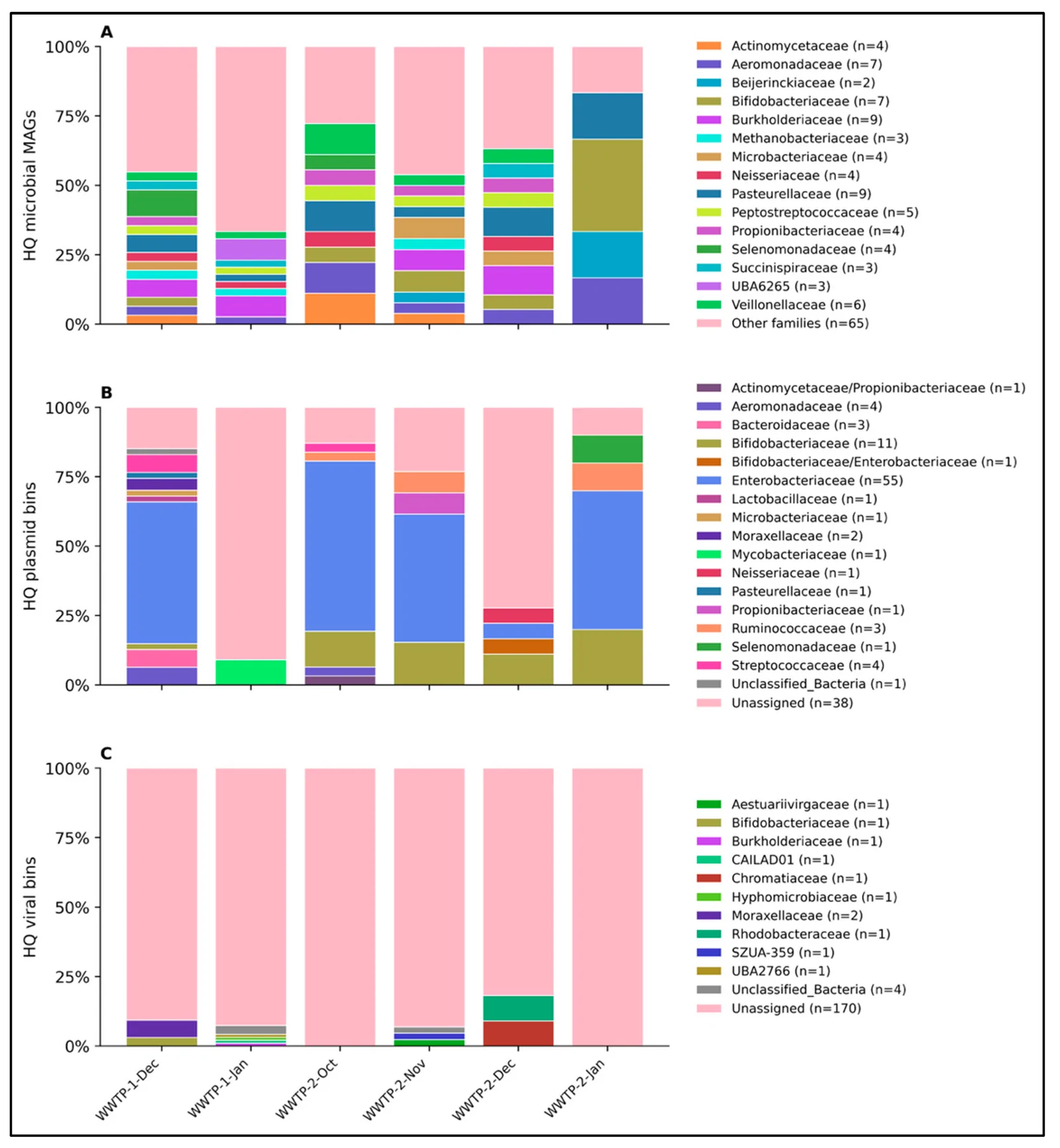
Family-level taxonomic composition of quality-filtered microbial MAGs, plasmid bins and viral bins across six wastewater samples. (**A**) GTDB family-level composition of microbial MAGs (*n* = 139). The 15 most abundant families are shown individually, with the remaining families grouped as other families. (**B**) Plasmid bins (*n* = 130) coloured by their Hi-C-assigned bacterial host family. Two plasmid bins associated with multiple bacterial host families are displayed as separate legend categories. (**C**) Viral bins (*n* = 185) coloured by their Hi-C-assigned bacterial host family. Bacterial families shared among panels are represented by the same colours. Unclassified_Bacteria and host-unassigned bins are shown in grey and light pink, respectively.

**Figure 3:**
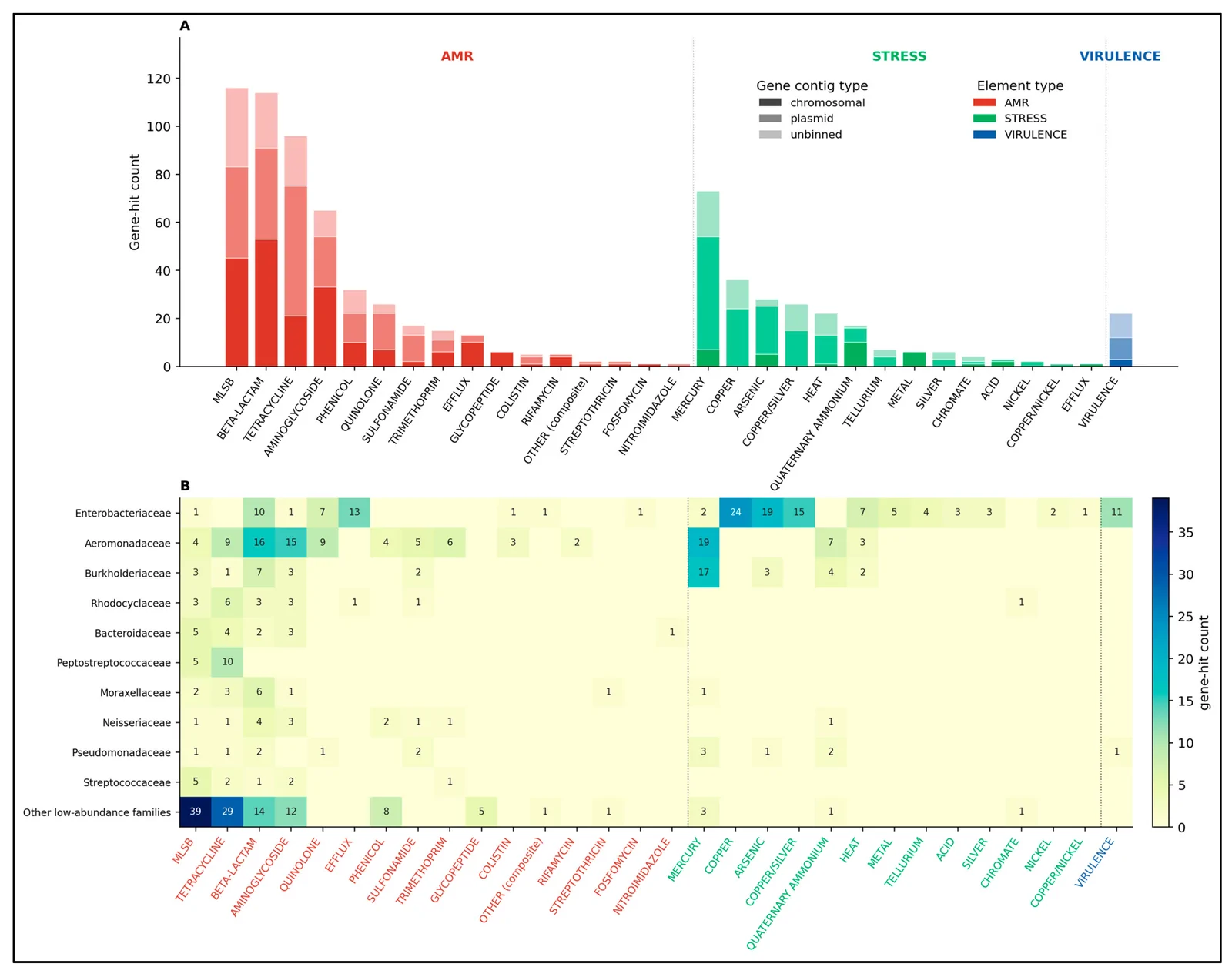
Distribution of antimicrobial resistance, stress-response and virulence determinants across contig types and bacterial host families. (**A**) Gene-hit counts for AMR, stress-response and virulence determinants grouped by gene class. Bars represent the total number of gene hits within each gene class, with stacked segments indicating contig type (chromosomal, plasmid and unbinned). Gene classes are grouped as AMR, stress response and virulence-associated. (**B**) Heatmap showing host-assigned gene hits across bacterial families and gene classes. Cell values and colour intensity indicate the number of gene hits assigned to each bacterial family–gene class combination. The ten bacterial families with the highest numbers of host-assigned gene hits are shown individually, whereas the remaining families are grouped as “Other families”. Bacterial families are ordered by total host-assigned gene-hit count, and gene classes are grouped by functional category.

**Figure 4:**
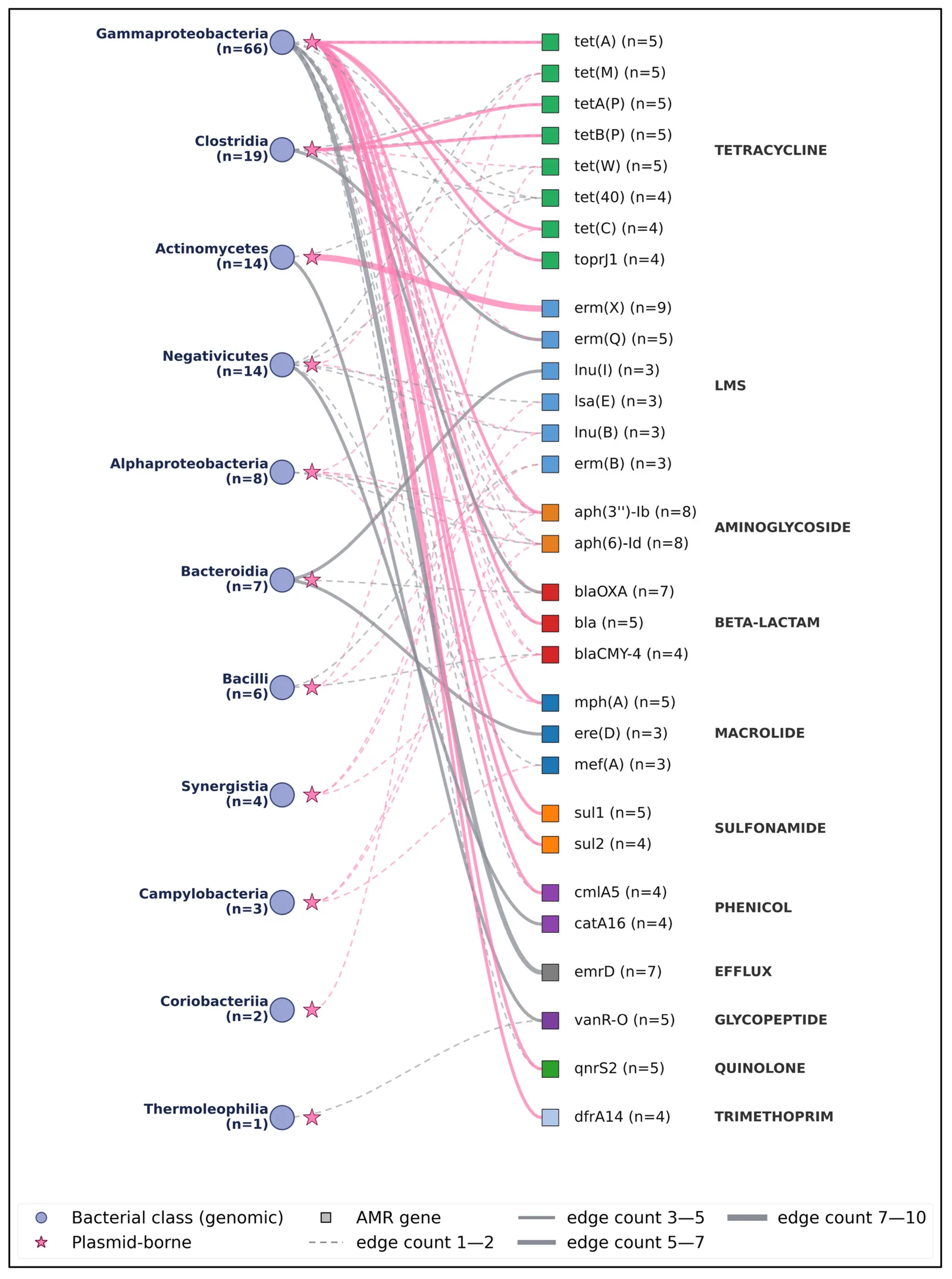
Gene-centred host–AMR association network of antimicrobial resistance determinants. Host classes are shown on the left and AMR genes on the right, grouped according to resistance class. Circles represent chromosomal detections and stars represent plasmid-associated detections. Node colour denotes bacterial host class, whereas AMR-gene nodes are coloured according to resistance category. Grey edges indicate bacterial host–ARG associations, whereas pink edges indicate plasmid-borne host–ARG associations. Edge thickness corresponds to the number of host–gene associations. Numbers in parentheses indicate the total number of detections for each host class or AMR gene. Unclassified and host-unresolved assignments were excluded from the analysis.

**Table 1:** Summary of microbial, plasmid and viral bin recovery, assembly size and sequencing depth across wastewater metagenomes.

Sample ID	Total Bins	Microbial MAGs (Quality-Filtered) *	Plasmid Bins (Quality-Filtered) **	Viral Bins (Quality-Filtered) ***	Binned Assembly (Mb)	Mapped Reads (×10^6^)
WWTP-1-Dec	4380	366 (31)	3406 (47)	607 (32)	1338.0	96.6
WWTP-1-Jan	2677	281 (39)	975 (11)	1420 (94)	1146.1	70.0
WWTP-2-Oct	1582	75 (18)	1481 (31)	25 (2)	394.5	13.3
WWTP-2-Nov	1972	147 (26)	1184 (13)	640 (43)	616.7	51.7
WWTP-2-Dec	1545	142 (19)	1225 (18)	177 (11)	523.4	9.4
WWTP-2-Jan	721	76 (6)	570 (10)	74 (3)	298.4	17.5
Total	12,877	1087 (139)	8841 (130)	2943 (185)	4317.1	258.6

Values are presented as total recovered bins, with the number meeting the specified high-quality thresholds shown in parentheses. * Microbial MAGs: ≥70% CheckM completeness and ≤10% contamination; ** Plasmid bins: ≥50% completeness and ≤20% contamination; *** Viral bins: ≥50% CheckV completeness and ≤20% contamination.

**Table 2:** Major bacterial reservoirs of AMR-positive plasmids identified by Hi-C linkage.

Family	Host–Plasmid Associations	Unique AMR-Positive Plasmid Bins	Representative Genera	Highest MAG Degree
Aeromonadaceae	30	28	*Tolumonas*	10
Enterobacteriaceae	19	17	*Klebsiella*, *Escherichia*	10
Bifidobacteriaceae	9	5	*Bifidobacterium*	2
Rhodocyclaceae	7	6	*Propionivibrio*	3
Neisseriaceae	7	7	*Neisseria*	6
Peptostreptococcaceae	6	6	*Romboutsia*	2
Pseudomonadaceae	6	6	*Pseudomonas*	4
Moraxellaceae	5	5	*Acinetobacter*	2
Streptococcaceae	5	5	*Streptococcus*	2
Bacteroidaceae	5	5	*Bacteroides*	2

Highest MAG degree indicates the maximum number of unique AMR-positive plasmids linked to a single MAG within each family.

## Data Availability

The raw sequencing data generated during this study are publicly available through the NCBI BioProject database under accession number PRJNA1496626.

## Supplementary Materials

The following supporting information can be downloaded at: https://www.mdpi.com/article/10.3390/microorganisms14102153/s1. Table S1: Plasmid contigs with Hi-C contacts to MAGs from two distinct bacterial classes.

## Abbreviations

The following abbreviations are used in this manuscript:

AMR	Antimicrobial resistance
ARG	Antimicrobial resistance gene
BWA-MEM	Burrows–Wheeler Aligner maximal exact matches
CFU	Colony-forming unit
DNA	Deoxyribonucleic acid
dsDNA	Double-stranded DNA
GC	Guanine–cytosine
GTDB	Genome Taxonomy Database
GTDB-Tk	Genome Taxonomy Database Toolkit
Hi-C	High-throughput chromosome conformation capture
MAG	Metagenome-assembled genome
Mb	Megabase
MGE	Mobile genetic element
MIMAG	Minimum Information about a Metagenome-Assembled Genome
MLSB	Macrolide–lincosamide–streptogramin B
N50	Assembly contiguity metric representing the contig length at which 50% of the assembly is contained in contigs of that length or longer
PCR	Polymerase chain reaction
ROC	Receiver operating characteristic
rRNA	Ribosomal ribonucleic acid
UAE	United Arab Emirates
vMAG	Viral metagenome-assembled genome
WBS	Wastewater-based surveillance
WWTP	Wastewater treatment plant
