# Supplementary material for: Resolving Host-Associated Antimicrobial Resistance in Municipal Wastewater Using Hi-C Metagenomics
Source: Microorganisms. 2026 Sep 24;14(10):2153. doi: 10.3390/microorganisms14102153 (PMC13707857; doi:10.3390/microorganisms14102153)
Supplement: Supplementary file 1 [file microorganisms-14-02153-s001.zip › microorganisms-4478192-supplementary.pdf]

Supplementary Table S1: Plasmid contigs with Hi-C contacts to MAGs from two distinct bacterial classes.

Sample	Contig	Gene(s)	Class 1	Class 2
WWTP-2-Oct	k99_11366	*tet(W)*	Clostridia	Coriobacteriia
WWTP-2-Nov	k99_420566	*aph(3″)-Ib*, *aph(6)-Id*	Gammaproteobacteria	Alphaproteobacteria
WWTP-2-Nov	k99_152708	*tet(C)*	Gammaproteobacteria	Alphaproteobacteria
WWTP-2-Dec	k99_417415	*aph(3″)-Ib*, *aph(6)-Id*	Campylobacteria	Alphaproteobacteria
WWTP-1-Dec	k99_1341003	bla_{CMY-4}	Clostridia	Synergistia

All contigs are plasmid-type. Each contig was Hi-C linked to one MAG from each of the two listed classes within the same sample. MAG IDs and full genome identifiers are provided in the supplementary data repository
